# Consensus on the treatment of alopecia areata – Brazilian Society of Dermatology^⋆^^⋆⋆^

**DOI:** 10.1016/j.abd.2020.05.006

**Published:** 2020-10-08

**Authors:** Paulo Müller Ramos, Alessandra Anzai, Bruna Duque-Estrada, Daniel Fernandes Melo, Flavia Sternberg, Leopoldo Duailibe Nogueira Santos, Lorena Dourado Alves, Fabiane Mulinari-Brenner

**Affiliations:** a Department of Dermatology and Radiotherapy, Universidade Estadual Paulista, Botucatu, SP, Brazil; b Department of Dermatology, Universidade de São Paulo, São Paulo, SP, Brazil; c Centro de Estudos dos Cabelos, Instituto de Dermatologia Prof. Rubem David Azulay, Santa Casa da Misericórdia do Rio de Janeiro, Rio de Janeiro, RJ, Brazil; d Department of Dermatology, Universidade do Estado do Rio de Janeiro, Rio de Janeiro, RJ, Brazil; e Department of Dermatology, Universidade Federal de São Paulo, São Paulo, SP, Brazil; f Department of Medicine, Santa Casa de Misericórdia de São Paulo, São Paulo, SP, Brazil; g Department of Dermatology and Allergology, Hospital do Servidor Público Municipal, São Paulo, SP, Brazil; h Department of Tropical Medicine and Dermatology, Universidade Federal de Goiás, Goiânia, GO, Brazil; i Department of Clinical Medicine, Universidade Federal do Paraná, Curitiba, PR, Brazil

**Keywords:** Adrenal cortex hormones, Alopecia areata, Consensus, Methotrexate, Therapeutics

## Abstract

**Background:**

Alopecia areata is a highly frequent disease with an impact on quality of life and several treatment options with little clinical confirmatory evidence.

**Objective:**

To disseminate the recommendations of Brazilian dermatologists with expertise in the treatment of alopecia areata.

**Methods:**

Eight specialists with expertise in alopecia areata from different university centers were appointed by the Brazilian Society of Dermatology to reach a consensus on its treatment. Based on the adapted DELPHI methodology, the relevant elements were considered; then, an analysis of recent literature was carried out and the consensus was written down. Consensus on the management of alopecia areata was defined with the approval of at least 70% of the panel.

**Results/Conclusions:**

Intralesional injectable corticotherapy was considered the first option for localized disease in adults. In extensive cases with signs of activity, systemic corticosteroid therapy should be considered and can be used together with immunosuppressants (corticosteroid-sparing agents). The use of an immunosensitizer (diphencyprone) is an option for stable long-term cases. Evaluation of side effects is as important as the rate of hair regrowth.

## Introduction

Alopecia areata (AA) is an autoimmune disease that targets hair follicles in the anagen phase and causes non-scarring alopecia. AA usually manifests before the age of 40 years, with no predilection for sex or race. The lifetime risk of developing AA is estimated at 2%.^1^ In a survey conducted by the Brazilian Society of Dermatology, AA accounted for 1.2% of all dermatological consultations. Among the causes of hair loss, it was only less frequent than androgenetic alopecia and telogen effluvium.^2^

Most studies on AA treatment do not present a high level of scientific evidence.^3^ This study aims to guide the treatment and follow-up of patients with AA based on the recommendations of a group of Brazilian specialists.

## Methods

Eight specialists with expertise in AA from different university centers were appointed by the Brazilian Society of Dermatology to reach a consensus on its treatment. The adapted DELPHI methodology was used.

The first step was to define the structure of the text and the topics to be addressed. Then, the themes were divided among the participants, who carried out a bibliographic review and drafted the text. Subsequently, all sections and recommendations on the treatment of AA were reviewed and discussed among all members. Consensus was defined as approval by at least 70% of the panel.

## Pre-treatment considerations

The pre-treatment considerations are summarized in Table 1.

**Table 1:** Considerations prior to the treatment of alopecia areata.

• Chronic disease of unpredictable course.
• Affection of large areas, long evolution, and early age onset are associated with a worse prognosis.
• Complementary tests are not mandatory for diagnosis and evaluation of comorbidities.

#### Clinical presentation

According to the extent and topography of involvement, AA is clinically classified into different patterns, as shown in Table 2.^4^, ^5^, ^6^

**Table 2:** Clinical forms of alopecia areata (AA).

Patch AA	Circumscribed areas of alopecia, called patches, oval or rounded; single or multiple.
Total AA	Alopecia affecting the entire scalp.
Universal AA	Alopecia affecting the entire body.
Ophiasis AA	Alopecia in the lateral and occipital regions of the scalp.
Inverted ophiasis AA (sisaipho)	Alopecia in the frontal-parietal area, sparing the lateral and occipital regions.
Diffuse AA	Overall decrease in density, without patches.
Diffuse acute and total	Diffuse, progressive and rapid hair loss, usually progressing to total AA within three months.

In order to determine the different types of treatment for each patient, this consensus considered AA as severe when it affected more than 50% of the scalp or when it affected over 25% of the scalp and presented a rapid evolution or had high impact on patient's quality of life.

#### Prognosis

AA is a chronic disease of unpredictable course. One may face spontaneous remission or progress to extensive hair loss not responding to any treatment.^4^ About 50% of patients with AA experience spontaneous repilation in the first six months and 70% present repilation in the first year, although AA may recur months or years after remission.^7^

Extensive forms of the disease generally do not respond well to treatment. Approximately 7% of the patients evolve to the subtypes of total alopecia (TA) or universal alopecia (UA).^7^ The long-term recovery rates of TA and UA are less than 10%.^7^ Patients with the “diffuse acute and total” subtype have a favorable prognosis, regardless of treatment.^6^ The factors associated with a worse prognosis are summarized in Table 3.^7^, ^8^, ^9^

**Table 3:** Factors for poor prognosis in alopecia areata (AA).

• Childhood disease onset.
• Episode duration greater than 1 year.
• Extensive area or ophiasis AA.
• Nail involvement.
• Association with atopy.
• Association with autoimmune diseases, mainly endocrine.
• Family history of AA.
• Association with genetic diseases, such as Down syndrome.

In this consensus, an acute episode was considered to be one that lasted up to six months and a chronic episode, one lasting more than six months. It is not known whether rapid treatment of the acute episode decreases the chance of developing chronic disease.^8^ The presence of signs of activity (Table 4) helps in the therapeutic decision. The time interval between episodes is unpredictable, and they may not even happen again. Permanent maintenance treatment is not recommended to prevent relapse.^8^

**Table 4:** Activity criteria for alopecia areata.

**Clinical**
Increased size or appearance of new patches.
Positive traction test.
**Dermatoscopic**
Black dots.
Exclamation mark hairs.
Broken hair shafts.
Pohl-Pinkus constrictions.

#### Complementary exams

The diagnosis of AA is made based on clinical and dermoscopic evaluation, and anatomopathological examination can be performed in doubtful cases. Complementary exams are not mandatory. Depending on the clinical suspicion, blood count, fasting blood glucose, FAN, VDRL, TSH, free T4, anti-TPO, antithyroglobulin, 25-OH vitamin D, vitamin B12, zinc, ferritin, and C-reactive protein may be requested.

#### Psychosomatic aspects

A proper conversation with the patient with AA starts by discussing their emotional aspects, including triggering factors and chances of hair regrowth. The psychological and social impact of hair goes beyond its biological significance. The negative effects of AA on the social and emotional well-being and on mental health were evidenced by quality of life indexes. To consider AA as mere hair loss does not take into account the negative impact that the disease can have on the patient's life.

A patient’s self-image, interpersonal relationships, and work or school activity can be affected by AA, even in cases with localized disease. More than half of the patients believe that the disease has major consequences in their lives.^10^ Acknowledging the emotional aspects of this “cosmetic” disorder allows comprehensive patient care.^11^

Psychiatric diagnoses such as depression, anxiety disorder, adjustment disorders, and paranoid disorders have been reported in up to 78% of patients.^12^, ^13^ AA is the second leading cause of psychiatric referral by dermatologists, second only to psoriasis.^14^

The isolated effectiveness of antidepressants, psychotherapy, relaxation techniques, and individual or group therapy in the treatment of AA has not been evaluated by clinical trials.^11^ Hypnotherapy has demonstrated improvement in hair growth and in the quality of life index in dermatology, with varied responses and high recurrence.^15^ More than half of patients with AA believe that their behavior could determine the improvement or worsening of the disease.^10^ To understand and respond to the difficulties that chronic AA can present, patients create their own models of their condition. Information received from various sources, including doctors, family, friends, the internet, and existing social and cultural notions about health and illness shape this model. These beliefs motivate treatment. If the belief system is inadequate, low compliance and treatment abandonment are frequent.^11^

Support groups can play a crucial role in coping with AA. Group partners help patients find their own identity. Sharing experiences with others with the same disease can be a fundamental link for treatment. Parents should always be the focus of pediatric AA. They can send their children the message that they are not “normal” or “beautiful”. Support groups help parents learn to communicate to their children that they are special and equal to them, with or without hair. Regular meetings of AA patients and family members can be an invaluable resource. In these groups, positive strategies for coping with daily difficulties such as the application of prostheses and uncomfortable situations are shared, in addition to updates on research with a view to the future of the disease. The psychosomatic aspects are summarized in Table 5.

**Table 5:** Psychosomatic aspects in alopecia areata.

• Understanding the negative psychosocial consequences and identifying patients who need psychological support is the role of the dermatologist.
• Cooperation between doctors and psychotherapists has a positive effect on the treatment and quality of life of the patient.
• Information and explanations are the best strategies to increase adherence to treatment.
• Support groups should be encouraged.

### Treatment

One of the essential steps in the treatment of AA is explaining to the patient the nature and course of the disease, as well as the available therapies, to realistically discuss the patient’s expectations.^4^ Due to the variable efficacy of treatments and their respective side effects, clinicians play an important role, making the patient more aware of the positive and negative aspects of each option. It should be clear to the patient that none of the therapies has been proven to change the course of the disease in the long term.^4^ AA treatment is not mandatory; therefore, the decision must be shared with the patient.^1^ Going untreated is a legitimate option for many patients.^8^ Table 6 summarizes the therapeutic considerations.

**Table 6:** Therapeutic approach to alopecia areata.

• There is no evidence that treatment changes the course of the disease in the long run.
• The assessment of side effects is as important as the rate of therapeutic response.
• No treatment is an option to be considered.

#### Intralesional corticosteroid therapy

This route of administration overcomes the epidermal barrier, making the drug available directly in the inflamed area.^16^, ^17^, ^18^ Thus, it minimizes the possible adverse effects of systemic corticosteroid (CT) therapy, with greater drug penetration when compared with the topical route.^18^ Patients with isolated alopecia patches, of small size (<3 cm), short duration, or occupying less than 25% of the scalp are the best candidates for intralesional infiltration.^5^, ^16^, ^19^ To date, no randomized clinical studies attested the effectiveness of this therapeutic modality.^20^

Approximately 60%–75% of patients with AA in patches who undergo CT infiltration present hair growth, a rate that varies with the severity of the disease; the perception of the clinical response generally occurs six weeks after the beginning of the treatment.^19^, ^21^, ^22^ Intralesional corticotherapy can be applied on the scalp, eyebrows, beard, and other affected body areas.^5^, ^16^ Although it is an important pillar of AA treatment, there is still no consensus regarding the ideal concentration and total dose of the drug.^23^

Triamcinolone acetonide (TAc) is the most widely used injectable synthetic CT worldwide.^4^, ^24^ In Brazil, TAc is restricted to intraocular use and, therefore, the most widespread form has become triamcinolone hexacetonide (TH), its less soluble derivative and with a higher risk of cutaneous atrophy.^18^ Classically, the concentration of 2.5–10 mg/mL of triamcinolone is used for the scalp and 2.5–5 mg/mL for the face and other body areas.^3^, ^25^ A pilot study showed that lower concentrations, such as 2.5 mg/mL, were as effective as higher concentrations and with a lower risk of atrophy.^26^ An alternative option to TH for the intralesional treatment of AA is betamethasone (betamethasone dipropionate 5 mg/mL + disodium phosphate betamethasone 2 mg/mL).^18^ A good response has also been reported with hydrocortisone acetate 25 mg/mL.^16^

The technique consists on infiltration of 0.05−0.1 mL per puncture into the dermis or upper part of the subcutaneous tissue, with a spacing of 0.5–1 cm between the punctures and an interval of four to six weeks between sessions.^4^, ^8^, ^21^ Dilution with saline or glycoside is recommended, and it may or may not be mixed with lidocaine.^18^ If the anesthetic has methylparaben, propylparaben, or phenol as components of its vehicle, the addition of lidocaine can increase the risk of flocculation of the drug, which can increase the chance of atrophy.^18^

Pain is a limiting factor, especially in children or patients with extensive forms of AA.^4^, ^22^ The use of topical anesthetics, vibration, or local cooling before application can be useful to minimize the discomfort of the procedure.^18^, ^27^ Needle-free devices are also an option; the device must be sterilized before use.^4^ These complementary measures may enable infiltration in children and even in adults who, at first, reject intralesional therapy. Treatment should be suspended if no improvement is observed six months after the beginning of the infiltrations.^4^, ^20^ Patients who do not respond to glucocorticoids may show resistance due to the low expression of thioredoxin reductase 1 in the external root sheath of the hair follicle.^20^, ^24^

Adverse effects include pain and hemorrhage at puncture sites, headache, reversible local cutaneous atrophy, dyschromia, systemic absorption and, much more rarely, anaphylaxis.^8^, ^17^ Atrophy is common and can be minimized by applying small volumes of the drug, using more diluted concentrations, increasing the spacing between punctures, and avoiding superficial injections outside the correct drug administration plane.^20^ Increased intraocular pressure and cataracts have already been reported in patients undergoing multiple sessions of intralesional CT therapy for AA in the eyebrow.^16^ The considerations about intralesional CT therapy are summarized in Table 7.

**Table 7:** Intralesional corticosteroid therapy in alopecia areata.

• First option for adults with localized disease.
• Better response in cases with signs of activity.

#### Topical corticosteroid therapy

Topical CT therapy is widely used in the treatment of all forms of AA, although its clinical efficacy is controversial, due to limited evidence.^4^ It is usually used isolated only in limited AA, since its effectiveness is apparently lower in more advanced forms of the disease.^19^ However, its use and possible benefits encompass all subtypes of AA, including in association with systemic therapy in extensive cases.

Different topical CTs have already been used in AA, with variable responses.^20^ Comparative studies, however, have shown that very high-potency CTs, such as clobetasol, are significantly more effective than others of lesser potency, such as hydrocortisone.^22^, ^28^ The advantages of this therapeutic modality include the lesser side effects in relation to the systemic route, greater patient compliance, and the possibility of use in several vehicles.^3^ Clobetasol has already shown a positive result when used as foam, cream, or ointment.^3^, ^21^ Its effectiveness appears to increase when applied under occlusion, with promising results even in severe forms of the disease, such as TA and UA, although it can increase the chance of side effects.^21^, ^24^

The most common adverse effects are folliculitis, local skin atrophy, striae, acneiform rash, telangiectasias, dyschromia, and rarely adrenal suppression.^4^, ^20^ Washing the site after 12 h of application is recommended to reduce the incidence of folliculitis and fractionizing the dose up to five times a week appears to prevent the onset of atrophy.^4^ The recommendations for topical corticotherapy are summarized in Table 8.

**Table 8:** Topical corticosteroid therapy in alopecia areata.

• Option for children or for patients who refuse intralesional therapy.
• It can be combined with other treatments.

#### Topical calcineurin inhibitors

Preliminary studies in animal models have suggested that tacrolimus may be a promising drug for the treatment of AA in humans. However, tacrolimus 0.1% ointment twice daily for six months was shown to be ineffective in inducing repilation in AA patients.^4^, ^29^ Despite the lack of literature data proving its effectiveness, topical tacrolimus is used almost as often as minoxidil for the treatment of AA, especially in the face region and in children.^29^

Superiority in the efficacy of pimecrolimus cream 1% when compared with placebo was not demonstrated.^30^ Similarly to tacrolimus, pimecrolimus does not appear to be a good therapeutic option for AA, especially in patients who are unresponsive to previous therapies. The recommendations on topical immunomodulators are summarized in Table 9.

**Table 9:** Calcineurin inhibitors in alopecia areata.

• There is no evidence of benefit.
• Routine use of topical calcineurin inhibitors is not recommended.

#### Topical minoxidil

The mechanism of action through which minoxidil stimulates the hair follicle has not been fully clarified. Vasodilation, angiogenesis, opening of potassium channels, and stimulation of the proliferation of follicular dermal papilla cells are some of the proposed mechanisms.^31^, ^32^ Based on the premise that it can increase the duration of the anagen phase of the hair cycle, the application of minoxidil appears reasonable as soon as the repilation starts, in order to increase the thickness and length of the new hair.

A recent meta-analysis considered minoxidil 5% to be superior to placebo when used in AA in patches. This evidence was classified as having moderate quality.^33^ Concomitant use with topical anthralin or intralesional corticotherapy appears to provide superior results when compared with isolated treatments.^29^ Expected side effects are hypertrichosis, contact dermatitis, and pruritus.^20^

Albeit controversial, topical minoxidil has been widely applied in clinical practice as adjuvant therapy in AA. The usual concentration of use in adults is 5%, ranging from one to two daily applications. The recommendations on topical minoxidil are summarized in Table 10.

**Table 10:** Topical minoxidil in alopecia areata.

• Inconsistent results when on monotherapy.
• It can be used as an adjuvant, mainly in localized forms, with partial repilation.

#### Topical immunotherapy

Topical immunotherapy (TIT) is performed with agents that trigger allergic contact dermatitis. They are used in the treatment of AA in order to decrease lymphocytic inflammation of the anagen follicle. The mechanism is not well understood, but it is believed to be related to deviation of the inflammatory process from the bulb to the place where the new inflammatory process is being induced by the medication. In this way, the follicle is able to recover.^34^, ^35^

Upon contact with the patient's skin, the sensitizer triggers a delayed hypersensitivity reaction (type IV), originating memory lymphocytes. Thus, after new exposure, the immune system will produce an inflammatory response mediated by T cells.

In Brazil, the sensitizing agent most commonly used is diphencyprone (DPCP), available only at compounding pharmacies. It must be formulated in acetone (highly volatile) and kept in containers protected from light (amber bottle).

After initial exposure to DPCP, the entire immune cascade takes between two to three weeks to elicit memory. Only after this period, the substance will be recognized as an allergen, causing dermatitis.

DPCP is indicated mainly in extensive cases. The rate of satisfactory clinical response can vary between 30%–48%; the rate of any type of response can reach up to 72.2%.^34^, ^36^, ^37^ The use of this substance is divided into three phases: initial, follow-up, and maintenance. Each phase has its peculiarities, which will be discussed below:

##### Phase I (initial or sensitizing)

In this phase, 2% DPCP diluted in acetone is used. A small amount is applied with a soaked cotton swab, in an area of 2 × 2 cm. The sensitization process can cause local hypo- or hyperchromia, which is why it is suggested that the application be done in a barely visible area, such as the scalp behind the ear. It is recommended not to expose the area to the sun or wash it for 48 h.

##### Phase II (follow-up)

Two to three weeks after Phase I, Phase II can begin. In this step, DPCP is applied weekly at low concentration, which is gradually increased. This is the most delicate moment of TIT, since it is necessary to establish the lowest concentration of DPCP that causes an inflammatory process on the skin. The application should not be started with the same concentration as the initial phase, as there is a great chance of an exacerbated inflammation, leading to complications such as blisters.

The medication should be applied to half of the scalp enough to slightly moisten the skin. One end of a soaked cotton swab is enough to spread the medication over one half of the scalp.

Thus, it is suggested to start with low concentrations, to be progressively increased every two weeks: 0.01%, 0.02%, 0.05%, 0.1%, 0.2%, 0.5%, 1%, and 2%. The speed of progression is at the physician's discretion; it can be even slower, using intermediate concentrations between those recommended. The ideal concentration should induce mild to moderate erythema, scaling, itching, and discomfort in the first 48 h after application; meaning that it was strong enough to trigger an inflammatory process. Thus, if the patient does not report any signs/symptoms in two applications, it is possible to increase the concentration in the third session.

After reaching the ideal concentration, treatment should be continued weekly. In cases of lack of response for six months, another treatment can be attempted. However, some authors report the need to persist with treatment up to 12–24 months to observe hair regrowth.^37^ In case of hair regrowth, treatment must be maintained until acceptable cosmetic coverage is reached and no signs of activity are observed.

##### Phase III (weaning/maintenance)

After cosmetic hair regrowth, it is suggested to reduce the frequency of applications to biweekly, followed by monthly and, finally, suspension. In cases of disease recurrence after the medication is discontinued, fortnightly or monthly treatment maintenance should be considered.

The most frequent complications are hyperpigmentation, hypopigmentation, intense eczema, blisters, lymph node enlargement, folliculitis, and flu-like symptoms.^36^ TIT recommendations are summarized in Table 11.

**Table 11:** Topical immunotherapy in alopecia areata (AA).

• Extensive AA with no signs of activity.
• Localized AA, refractory to local corticosteroid therapy.
• Adults or children with the ability to properly perform treatment.

#### Anthralin

The use of anthralin also aims to divert the inflammatory process away from the bulb, but it causes irritative contact dermatitis, rather than the allergic form. In Brazil, anthralin is only available in compounding pharmacies. Its concentration can vary from 0.5% to 2.0% in lanette cream or solution.

Its use in children is justified by the lack of systemic side effects. Hair regrowth is observed in 71% of the cases of alopecia in patches.^38^ The mean time to initial response is three months, and a complete response is observed in 15 months.^38^

The drug is applied to the affected area, extending up to 1 cm into the apparently healthy area. The patient is instructed to wash the area and thoroughly remove the product after 30 min. The procedure is repeated daily. Every three days, the time of contact with the skin is increased by 15 min. That is, the initial time is 30 min, then increased to 45 min, 60 min, 75 min, and so on until the maximum time of two hours is reached. The goal is to cause mild eczema. When there is no reaction after two hours, the contact time can be extended, in some cases, to overnight.

**Figure 1:**
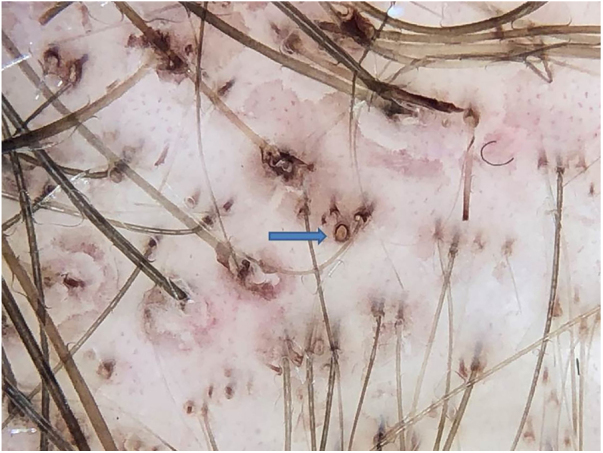
Brown pigmentation at the edge of the follicular ostium by anthralin (blue arrow); differentiate from the black dots that are in the center of the ostium.

Anthralin is a dark brown substance with a characteristic odor. With frequent applications, it ends up pigmenting the follicular ostia, simulating black dots. However, on dermoscopy, the difference between a black dot in the center of the ostium and the regularly distributed brown pigmentation on the edge of the follicular ostium by anthralin is clear (Fig. 1). One of the signs of therapeutic failure is evidenced by the lack of pigmentation of the ostia, which can have three main causes: irregular application, short contact time with the scalp, or low quality medication.

This is a safe therapy, but care must be taken to avoid complications. As the goal is to cause local irritation, the increase in the time of contact of the medication with the skin must be systematic, avoiding an exacerbated irritation.

The use of anthralin leads to hyperpigmentation of the treated area, but the color will return to normal after treatment discontinuation. The recommendations on anthralin are summarized in Table 12.

**Table 12:** Anthralin in alopecia areata (AA).

• Option for children due to the absence of systemic side effects.
• Second- or third-line treatment for adults.
• In association, it may be an option for extensive AA.

#### Systemic corticosteroids

Several forms of systemic administration of CTs have been described for the treatment of AA. A 200 mg oral prednisolone pulse once a week for three months presented a response rate of 40% in patients with more than 40% of the affected scalp or more than ten patches on the scalp or body.^39^ Other regimens include prednisolone 80 mg/day for three consecutive days every three months and for patients resistant to other therapeutic modalities, a megapulse of 15 mg/kg for two days every three weeks.^40^, ^41^

There is no consensus in the literature on the dose and duration of daily oral corticotherapy use in AA. Prednisone can be used in doses ranging from 0.1 to 1 mg/kg/day. It is suggested to start with higher doses (0.5–1 mg/kg/day), with a gradual reduction (over 6–12 weeks) after hair regrowth is achieved.

Although not described in guidelines for AA and presenting a higher cost, this consensus considers deflazacort to be the best option for oral CT, due to its more favorable safety profile. The drug has a high therapeutic index, with potency ranging between 70%−90% of prednisone, with a lower impact on calcium metabolism when compared with any other synthetic CT. In addition, it has comparatively little effect on carbohydrate metabolism, water retention, and hypokalemia.^42^ It is suggested to start with a daily dose of 0.5 mg/kg for both adults and children, with a slow reduction after hair regrowth.

Long-term CTs can be administered intramuscularly or intravenously as an alternative to oral use. In a comparative randomized clinical trial, the application of 40 mg of intramuscular TAc once a month for six months was superior to pulsed prednisolone (80 mg for three consecutive days every three months) and to dexamethasone (0.5 mg/day for six months).^40^

Similarly to the intralesional use, TAc is recommended over TH for intramuscular use, due to the greater risk of developing atrophy and telangiectasias in the long term.^43^ The authors suggest, as a second option, the substitution by the combination of betamethasone dipropionate 5 mg/mL (fast action) with disodium phosphate betamethasone 2 mg/mL (prolonged action).^18^ Table 13 presents the dose equivalence of the different CTs.^44^

**Table 13:** Equivalence of corticosteroids.

Substance	Equivalent dose (mg)	Anti-inflammatory potency	Mineralocorticoid potency	Half-life (h)
Cortisol (hydrocortisone)	20	1	1	8−12
Cortisone	25	0.8	0.8	8−12
Prednisone/prednisolone	5	4	0.8	12−36
Deflazacort	7.4	4	0.5	2
Methylprednisolone	4	5	0.5	12−36
Triamcinolone	4	5	0	12−36
Betamethasone	0.75	25	0	26−72
Dexamethasone	0.75	25	0	36−72
Fludrocortisone	_	10	125	12−36

Adapted from Caplan et al., 2017.^44^

The response rates with systemic CT are high, but many patients experience relapses with dose reduction or shortly after medication withdrawal. For patients who respond to CT, but who become steroid dependent, the combination of another systemic medication can be useful to spare the use of CT.

For all types of long-term CT therapy, it is important to classify and monitor the patient for the risk of side effects, in addition to providing information about interactions and vaccination.^44^, ^45^, ^46^, ^47^ The recommendations on systemic CT are summarized in Table 14.

**Table 14:** Systemic corticosteroids in alopecia areata.

• Indicated in disease with signs of activity and with extensive involvement.
• It is necessary to monitor possible short and long term side effects.

#### Immunosuppressants

##### Methotrexate (MTX)

MTX is a competitive chemotherapeutic inhibitor of dihydrofolate reductase. Its use in low doses in inflammatory diseases such as AA requires folic acid supplementation. Among the independent pathways, suppression of the JAK/STAT signaling pathway appears to be the main mechanism of action.^48^

Initial doses of 5–10 mg/week are progressively increased over four to six weeks, reaching up to 20–25 mg. As oral MTX in doses above 15 mg may present erratic absorption, the injectable option should be considered in these cases. Combination with oral and intralesional CT have been reported. In most cases, a minimum dose of MTX, ranging from 7.5 to 12.5 mg/week, is required for maintenance.^49^, ^50^

Better responses are observed in men, patients over 40 years old, those with less than five years of illness, those who reached cumulative doses of 1000−1500 mg, and those who received CT in addition to MTX.^50^, ^51^ Recurrence can happen during treatment and after discontinuation of medication. Prolonged use is necessary.

MTX in conjunction with low doses of prednisone showed hair regrowth of terminal hairs in up to 96% of patients with AA.^49^ Total hair regrowth has been demonstrated in 15%–64% of patients.^51^

Pancytopenia is the most common toxicity when using low-dose MTX. Patients with renal failure, hypoalbuminemia, those who use erroneously high doses, and those who use other drugs that interact with MTX (*e.g*., anti-inflammatory drugs) are at increased risk.^52^, ^53^ Interstitial pneumonitis and abnormal liver function have been reported.^51^ Folic acid supplementation reduces the side effects of MTX, especially gastrointestinal ones. Various doses and frequencies of administration have been described, ranging from 5 mg per week to 1–5 mg/day. Even in daily doses, folic acid does not interfere with the effectiveness of MTX.^54^ Folinic acid supplementation is reserved for cases of toxicity by the drug.^54^

##### Cyclosporine

Cyclosporine is an immunosuppressive agent capable of inhibiting the activation of auxiliary T cell and suppressing the production of gamma interferon, reducing the perifollicular inflammatory infiltrate. The high recurrence and high incidence of long-term side effects limit its use. This is the only immunosuppressant for which a controlled, double-blinded, randomized study demonstrated a response in AA.^55^

A daily dose of 2 mg/kg/day, divided into three intakes, is used initially, with a progressive increase of up to 5 mg/kg/day.^56^ Regrowth ranges from 25% to 76.6% when associated with systemic and intralesional CT.^56^, ^57^

Its use is limited by the high recurrence rates after withdrawal and by its side effects, especially nephrotoxicity, immunosuppression, and arterial hypertension. The reported nephrotoxicity, usually due to prerenal vasoconstriction, was reversible in patients with AA.^56^, ^57^

##### Azathioprine

Azathioprine is an antimetabolite with few reports of use in AA. Initial doses of 0.5–1 mg/kg/day can be increased up to 2–3 mg/kg/day, according to the patient's tolerance.^58^ A dose of 2.5 mg/kg/day can be considered for recalcitrant AA. In 43% of patients, some hair regrowth was observed after four to six months.^58^ Gastrointestinal symptoms, elevated liver enzymes, pancreatitis, and bone marrow suppression are the most common side effects.

Better responses can be observed in association with systemic/injectable CT or even MTX. The association with MTX should be carried out carefully, considering the increase in side effects.^59^ The recommendations on immunosuppressants are summarized in Table 15.

**Table 15:** Immunosuppressants in alopecia areata (AA).

• The use of immunosuppressants in AA has grown in the past 20 years.
• These drugs have been progressively included in the dermatologist's routine from experience in uncontrolled studies.
• Methotrexate is the best option, considering the need for long-term safe use.
• Cyclosporine should have its use limited to short periods, due to side effects; it is the only drug in the group evaluated in randomized studies.
• When used in isolation, azathioprine did not present a good response.
• Despite the side effects, this group of drugs must be considered in the therapeutic decision shared with the patient in cases of extensive disease.
• The association with systemic and intralesional corticosteroids enhances the results.

#### Janus kinase (JAK) inhibitors

Currently, the pathophysiology of AA is considered to be based on CD8+ cytotoxic T cells that express NKG2D. These lymphocytes are responsible for initiating and maintaining the autoimmune inflammation process against the hair follicle and are activated and perpetuated by IFN-γ and IL-15, which in turn use the JAK-STAT signaling pathway.^60^

JAK inhibitors are small molecule drugs approved for use in myelofibrosis, polycythemia vera, essential thrombocytosis, and rheumatoid and psoriatic arthritis, and have been studied in patients with AA refractory to other treatments.

Ruxolitinib, a JAK1 and JAK2 inhibitor, was investigated in 12 patients with moderate to severe AA (mean SALT: 65.6) at a dose of 20 mg twice daily for three to six months of treatment, and followed-up for three months after drug withdrawal. Nine of the 12 patients (75%) showed improvement, with an average hair regrowth of 92%, without serious adverse effects. Three months after the end of treatment, all patients had recurrence of hair loss.^61^ Other case reports have also been published.^62^

Tofacitinib inhibits JAK1/2 and more intensily, JAK3. It is the JAK inhibitor with the most cases published in the literature in various treatment regimens, such as 5 mg twice a day, increasing the dose to 10 mg twice a day and, if there is no response, association with prednisone 300 mg once a month.^63^, ^64^, ^65^ A recent meta-analysis included 14 studies, six clinical trials, and eight observational studies, with 275 patients. The rate of acceptable or complete repilation was 54% (95% CI: 46.3%−61.5%), and the partial response rate was 26.1% (95% CI: 20.7%−32.2%). During follow-up, approximately one-quarter of the patients presented recurrence after discontinuation.^66^

In general, the response to treatment with JAK inhibitors did not correlate with demographic characteristics, disease severity, and duration of AA, and there are no predictors of response to date.^67^ The most common complications are mild infections, mainly of the upper respiratory tract and urinary tract. Dyslipidemia, leukopenia, increased liver enzymes, headache, gastrointestinal complaints, fatigue, acne, and weight gain have also been reported. To date, there are no reports of neoplasms, reactivation of tuberculosis, or hospitalization due to adverse events. Long-term safety data is still limited.^67^

Topical JAK inhibitors are not yet available in standard formulations; the reports in the literature are based on compounded presentations, with variable results, and are currently not recommended.^67^

Treatment with JAK inhibitors is still based on low-quality evidence, predominantly case reports, retrospective studies, and non-blinded and non-placebo-controlled clinical trials. The development of potentially more selective JAK inhibitors and the optimization of topical formulations are promising. Recommendations regarding the use of JAK inhibitors are summarized in Table 16; Table 17 lists the pre-treatment evaluation.

**Table 16:** JAK inhibitors in alopecia areata.

• Ruxolitinib and tofacitinib have been shown to be effective in patients refractory to other treatments.
• High recurrence rates after suspension.
Generally mild side effects; however, there is still little data on long-term safety.
• Use limited by high cost.

**Table 17:** Recommended evaluation before starting treatment with JAK inhibitors.

• Not indicated in pregnant and lactating women.
• Assess the risk of cancer.
• Check drug interactions.
• Pre-treatment tests: complete blood count, kidney function, transaminases, and bilirubin (repeat every two weeks or more); lipidogram (repeat every three months).
• Take the recommended vaccines.
• Serology for hepatitis B, C and HIV.
• QuantiFERON or PPD for tuberculosis.
• Chest X-ray.
• Treatment of latent tuberculosis, if indicated.

### Other treatments

The treatments mentioned below have little evidence of results and should only be considered in the absence of response to standard therapies.

#### Hydroxychloroquine

An antimalarial with anti-inflammatory action and immunomodulatory effect. Studies on hydroxychloroquine in AA are controversial.^68^, ^69^ When used, the recommended dose is 5 mg/kg/day; in case of prolonged use of the drug, special attention should be paid to ocular toxicity. Gastrointestinal intolerance and headache are the most commonly reported side effects. Hydroxychloroquine is not recommended for the treatment of AA.

#### Zinc

A randomized study including patients with AA in patches, double-blinded with cross-over of patients using zinc sulfate at a dose of 5 mg/kg/day, divided into three doses, demonstrated the superiority of zinc over placebo.^70^ In another placebo-controlled study, including patients with TA and UA, no improvement was observed when comparing the treatment group with the control group.^71^ The authors consider that zinc sulfate could be used in less severe cases and, preferably, associated with other therapies. It can be used even if the patient presents normal serum zinc levels.

#### Sulfasalazine/mesalazine

Sulfasalazine is a prodrug composed of 5-aminosalicylic acid (5-ASA) associated with sulfapyridine. While 5-ASA is responsible for the efficacy of sulfasalazine, sulfapyridine accounts for most of the side effects: headache, anorexia, nausea, and vomiting, which occur in 10%−45% of patients. Mesalazine contains only slow-release 5-ASA; for this reason, it is usually better tolerated than sulfasalazine. 5-ASA works both as an immunomodulator and immunosuppressant and is used in several autoimmune conditions such as ulcerative colitis, Crohn’s disease, and psoriasis. An uncontrolled open study demonstrated rates of hair regrowth with sulfasalazine (1.5 g 2 ×/day) between 25% and 68% in patients with refractory AA, alone or in combination with CT therapy, acting as a CT-sparing agent. However, the side effect profile remains a limiting factor.^72^ Recently, hair regrowth was reported with the use of mesalazine (15−30 mg/kg/day in two daily doses) associated or unassociated with topical oral CT or minoxidil/betamethasone in the treatment of five children and adolescents (2–17 years) with refractory and severe AA.^73^ Follow-up with G6PD, blood count, biochemistry, and hepatogram assessment are essential to monitor possible adverse effects.

#### Simvastatin/ezetimibe

Data on the efficacy of the combination of simvastatin 40 mg/ezetimibe 10 mg, 1 ×/day in the treatment of AA are limited and controversial.^74^, ^75^, ^76^, ^77^ Positive reports show hair regrowth rates and lower relapse in patients with recent onset and good prognosis for AA.^74^, ^75^

#### Oral minoxidil

The first report of the use of oral minoxidil in AA was in 1987, in monotherapy at a dose of 5 mg, twice a day, in 65 patients, including men and women. Satisfactory repilation was observed in 18% of patients.^78^ Despite the high dose, facial hypertrichosis was observed in only 17% of patients. Recently, the combination of minoxidil (2.5 mg/day for women and 2.5 mg twice a day for men) and tofacitinib (5 mg twice a day) has been shown to be positive, with a low incidence of side effects.^79^

#### Excimer laser/light

The most studied light-based therapies in AA are the excimer laser and excimer light at 308 nm, which have immunosuppressive properties, possibly by inducing T-cell apoptosis. A recent review included eight clinical studies and case reports, with a total of 94 treated individuals, with an efficiency of 36.9%−100%, and hair regrowth equal to or greater than 50%.^80^ A meta-analysis that included only four controlled studies, using excimer laser on AA patches not previously treated, confirmed the effectiveness of the treatment.^81^ The main side effects of the therapy are mild erythema, pain during application, hyperpigmentation, blistering, pruritus, and peeling.^82^ The high cost is the main disadvantage of the treatment. Excimer laser and excimer light can be alternatives for refractory cases, especially when there is CT-induced atrophy or contact dermatitis by other therapies.

#### PUVA

Photochemotherapy with psoralen and ultraviolet A radiation (PUVA) has been investigated in several therapeutic schemes in studies from the 1980s to 1990s, with different efficacy rates. Due to the chronic and recurrent nature of AA, this therapeutic modality is not recommended due to the risk of large cumulative doses of UVA in the long term, with the possibility of cutaneous malignancy as an adverse effect. More recently, there has been interest in treatment with UVA-1, which has greater penetration into the skin and possibly greater effectiveness and may be an alternative in the treatment of AA.^83^

#### Dapsone

Due to its low efficacy and its side effect profile, there is no justification for the use of dapsone in AA.^84^, ^85^

#### Platelet-rich plasma (PRP) and microneedling

Small clinical trials have suggested a potential benefit of PRP and microneedling in AA, but the real benefit of these techniques has not yet been established.^86^, ^87^ The use of these modalities for the treatment of AA is not recommended. To date, the use of PRP for dermatological purposes is prohibited by the Brazilian Federal Council of Medicine.

#### Prostheses and camouflages

Cosmetic disguise options should be encouraged. Although few studies have quantified the benefit in the patients' quality of life, the suggestion of these resources is considered good practice.^88^, ^89^ There are numerous options for partial or total hair prostheses, and removable or fixed extensions. Camouflages can take the form of hair fibers, sprays, waxes, and pigmented powders. Trichopigmentation can be performed on the scalp and eyebrow area. Eyelashes, false nails, and eyebrow prostheses can also be used.

### Treatment in special situations

#### AA in children

The management of AA cases in children could even be considered an ordinary situation, as it is a common cause of hair loss in childhood and adolescence—up to 60% of cases start in the first two decades of life.^90^

In children, topical CT is used as the first therapeutic option, preferably of medium to high potency (mometasone 1% to clobetasol 0.05%). When there is contraindication or lack of response to topical CT, anthralin, DPCP, or minoxidil may be used, or an expectant treatment may be chosen.^38^, ^91^

The administration of systemic drugs can be considered in extensive cases or in cases with extensive disease activity. One should always take into account the child's life stage and degree of development. It is important to pay attention to possible adverse effects, especially changes in growth, and in metabolic and immune competence. Among the options available, there is a preference for systemic CT and MTX. Table 18 presents the main recommendations for the treatment of AA in children.

**Table 18:** Recommendations for the treatment of children with alopecia areata (AA).

• Localized AA: topical corticosteroid therapy (under occlusion, when possible) or anthralin 0.5%−1%, in short contact therapy.
• Extensive AA: systemic CTs orally or pulse therapy, and/or methotrexate 0.2−0.4 mg/kg/week, in cases with signs of activity. Diphencyprone immunotherapy in cases without signs of activity or with contraindication/lack of response to immunosuppressants.

#### AA in the beard area

The beard is the second most frequently affected area by AA, second only to the scalp.^5^ There are no controlled and randomized clinical trials evaluating treatment in this region. The most frequent approach is the use of local therapies, starting with topical CT followed by intralesional CT, or starting directly with the use of intralesional CT, a treatment that has the greatest evidence of efficacy for localized and short-term AA.^92^, ^93^ Triamcinolone concentration of 2.5 mg/mL is the most suitable for the treatment of the face. Other treatments described for the region include topical minoxidil, immunotherapy, photodynamic therapy, 1550 nm fractional erbium glass laser, PUVA, or narrow-band UVB phototherapy. Recommendations for treating the beard area are summarized in Table 19.

**Table 19:** Recommendations for the treatment of alopecia areata in the beard area.

• Consider not treating.
• Topical or intralesional corticosteroid therapy are the treatments of choice; however the risk of atrophy is greater in this area.
• Topical minoxidil and anthralin can be considered.

#### AA on eyebrows and eyelashes

The involvement of the eyebrows and eyelashes has a great impact on the quality of life, as it directly interferes with the facial appearance. The only agents indicated for hypotrichosis or alopecia in the eyelash region, regardless of their etiology, are prostaglandin analogues, especially the 0.03% bimatoprost solution.^94^, ^95^ Some studies have assessed the drug in adult and pediatric patients, with variable results, which is probably due to conditions with different severities and, therefore, different AA prognoses.^96^ It appears to be a safe and potentially effective agent for treating the region. It is important to note the risk of eyelid hyperpigmentation and darkening of the iris.

In the eyebrows, in addition to the possibility of using 0.03% bimatoprost, there is the option of topical and intralesional CT.^94^ Preferably, a medium-potency CT should be used, and care should be taken to prevent the drug from running into the eyelids and eyes (cream vehicle, instead of solution). Intralesional infiltration of triamcinolone should be done at a dose of 2.5 mg/mL to minimize the risk of cutaneous atrophy. Topical minoxidil is also an option, similarly to the scalp. Recommendations for the treatment of eyelashes and eyebrows are summarized in Table 20.

**Table 20:** Recommendations for the treatment of alopecia areata of eyebrows and eyelashes.

• Eyelashes: 0.03% bimatoprost solution, once daily. Use for one year to evaluate response.
• Eyebrows: topical corticosteroid of medium potency in cream (or high potency in alternate days) or intralesional triamcinolone 2.5 mg/mL. Alternatively 0.03% bimatoprost or topical minoxidil.

## Follow-up

The interval between follow-up appointments will depend on the treatment regimen chosen for each patient. Those treated with DPCP will require weekly or fortnightly visits; those submitted to intralesional infiltrations should be followed-up every four to six weeks. Patients undergoing topical home treatment can be assessed every two to three months, whereas those receiving systemic treatments will have intervals depending on the chosen drug and on the patient's health conditions. At the initial consultation and at least quarterly, standardized clinical photos documenting different regions of the scalp (top, sides, occipital region) should be taken. Dermoscopic photos of the areas of alopecia are recommended. The classification in one of the methods described for estimation of extension (SALT score, SALT II, ALODEX, Alopecia Areata Progressive Index) and assessment of nail involvement and the involvement of body hair must be included in the patient's medical record. Other instruments, such as quality of life questionnaires, can be adapted for use in AA.^97^

## Final considerations

**Figure 2:**
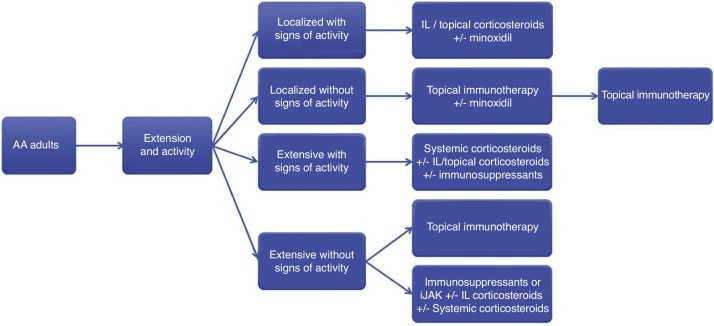
Algorithm for the treatment of alopecia areata in adults. AA, alopecia areata; IL, intralesional; iJAK, inhibitors of Janus kinase. Immunosuppressants: methotrexate, azathioprine and cyclosporine.

The treatment of AA is complex, and few comparative studies show confirmatory evidence. This consensus aimed to provide basic guidance for the management of AA in daily practice with a focus on the Brazilian reality. The recommendations presented herein are summarized in Fig. 2.

## Financial support

None declared.

## Authors’ contributions

Paulo Müller Ramos: Approval of the final version of the manuscript; design and planning of the study; elaboration and writing of the manuscript; critical review of the literature; critical review of the manuscript.

Alessandra Anzai: Approval of the final version of the manuscript; design and planning of the study; elaboration and writing of the manuscript; critical review of the literature; critical review of the manuscript.

Bruna Duque-Estrada: Approval of the final version of the manuscript, design and planning of the study; elaboration and writing of the manuscript; critical review of the literature; critical review of the manuscript.

Daniel Fernandes Melo: Approval of the final version of the manuscript; design and planning of the study; elaboration and writing of the manuscript; critical review of the literature; critical review of the manuscript.

Flavia Sternberg: Approval of the final version of the manuscript, design and planning of the study; elaboration and writing of the manuscript; critical review of the literature; critical review of the manuscript.

Leopoldo Duailibe Nogueira Santos: Approval of the final version of the manuscript; design and planning of the study; elaboration and writing of the manuscript; critical review of the literature; critical review of the manuscript.

Lorena Dourado Alves: Approval of the final version of the manuscript; design and planning of the study; elaboration and writing of the manuscript; effective participation in research orientation; critical review of the literature; critical review of the manuscript.

Fabiane Mulinari-Brenner: Approval of the final version of the manuscript; design and planning of the study; elaboration and writing of the manuscript; critical review of the literature; critical review of the manuscript.

## Conflicts of interest

None declared.
